# A case of sarcoidosis with isolated hepatosplenic onset and development of inflammatory bowel disease during recovery stage

**DOI:** 10.1007/s13317-017-0094-5

**Published:** 2017-04-28

**Authors:** Moris Sangineto, Chiara Valentina Luglio, Patrizia Suppressa, Carlo Sabbà, Nicola Napoli

**Affiliations:** Clinica Medica “Cesare Frugoni”, Department of Interdisciplinary Medicine, University of Bari “Aldo Moro”, Piazza Giulio Cesare 11, 70124 Bari, Italy

**Keywords:** Sarcoidosis, Liver, Spleen, Intestinal sarcoidosis, Crohn’s disease

## Abstract

Sarcoidosis is a systemic disease characterized by an immune-mediated disorder, which leads to the development of non-caseating granulomas in the involved organs. More than 90% of patients with sarcoidosis present lungs and lymphatic system involvement at onset, while less than 10% has an isolated extrapulmonary localization. Here, we describe the case of an elderly patient with isolated hepato-splenic onset (multiple splenic lesions at imaging and cholestasis), and subsequent pulmonary involvement. The liver biopsy showed the presence of non-caseating granulomas, suggesting sarcoidosis. Despite the complete recovery was obtained with steroid therapy, after dosage reduction the patient presented watery diarrhea. Endoscopic investigations with biopsies were performed, describing the presence of an important lympho-plasmacytic infiltrate of terminal ileum mucosa with typical aspects of inflammatory bowel disease. The symptomatology completely disappeared after steroid dosage increase. This case confirms that sarcoidosis could present in a very atypical way, involving several organs in a different manner at the same time and that every symptom should not be underestimated, despite the rare presentation.

## Introduction

Sarcoidosis is an immune-mediated disorder of unknown etiology, characterized by the presence of non-caseating granulomas in the involved organs [1, 2]. Mainly, it involves lungs and lymphatic system, but theoretically every tissue could be affected. Frequently involved organs are skin, eyes, peripheral lymph nodes and liver (10–25% of cases). Therefore, the clinical manifestations, as well as the disease evolution, are widely variable. The most serious complications are due to the pulmonary fibrosis, but also cardiac, renal, neurological, laryngeal and ocular localizations are responsible of severe manifestations [3].

## Case report

A 68-year-old female patient with a history of diabetes mellitus type 2 and hypothyroidism, presented with abdominal pain and pruritus. At admission, physical examination showed abdominal pain to palpation, involving epigastric and mesogastric area, and skin lesions by scratching. Laboratory findings showed erythrocyte sedimentation rate (ESR) 120 mm/h, C-reactive protein (CRP) 49.5 IU/mL, alkaline phosphatase (AP) 817 IU/mL, γ-glutamyl transferase (γ-GT) 1390 IU/mL, haemoglobin (Hb) 10.5 g/dL, carbohydrate antigen 19.9 (CA 19.9) 277.5 IU/mL. Other routine biochemical tests, including white blood cell (WBC) count, liver, pancreatic and renal function tests, serum immunoglobulins, blood and urinary protein electrophoresis and other tumoral markers were in the normal range. Abdomen ultrasonography (US) showed mild hepato-splenomegaly with liver steatosis and inhomogeneous echogenicity of the spleen because of the presence of multiple hypoechoic areas of varying sizes, the largest measuring 16 mm. Computerized tomography (CT) scan confirmed the presence of numerous splenic lesions, with perisplenic capsular calcifications, in the absence of other abdominal and/or thoracic pathological findings, except for liver steatosis. Mammography and breast US resulted negative.

Serum markers and stool microbiological examinations for viral, bacterial, fungal and parasite infections were negative.

**Fig. 1:**
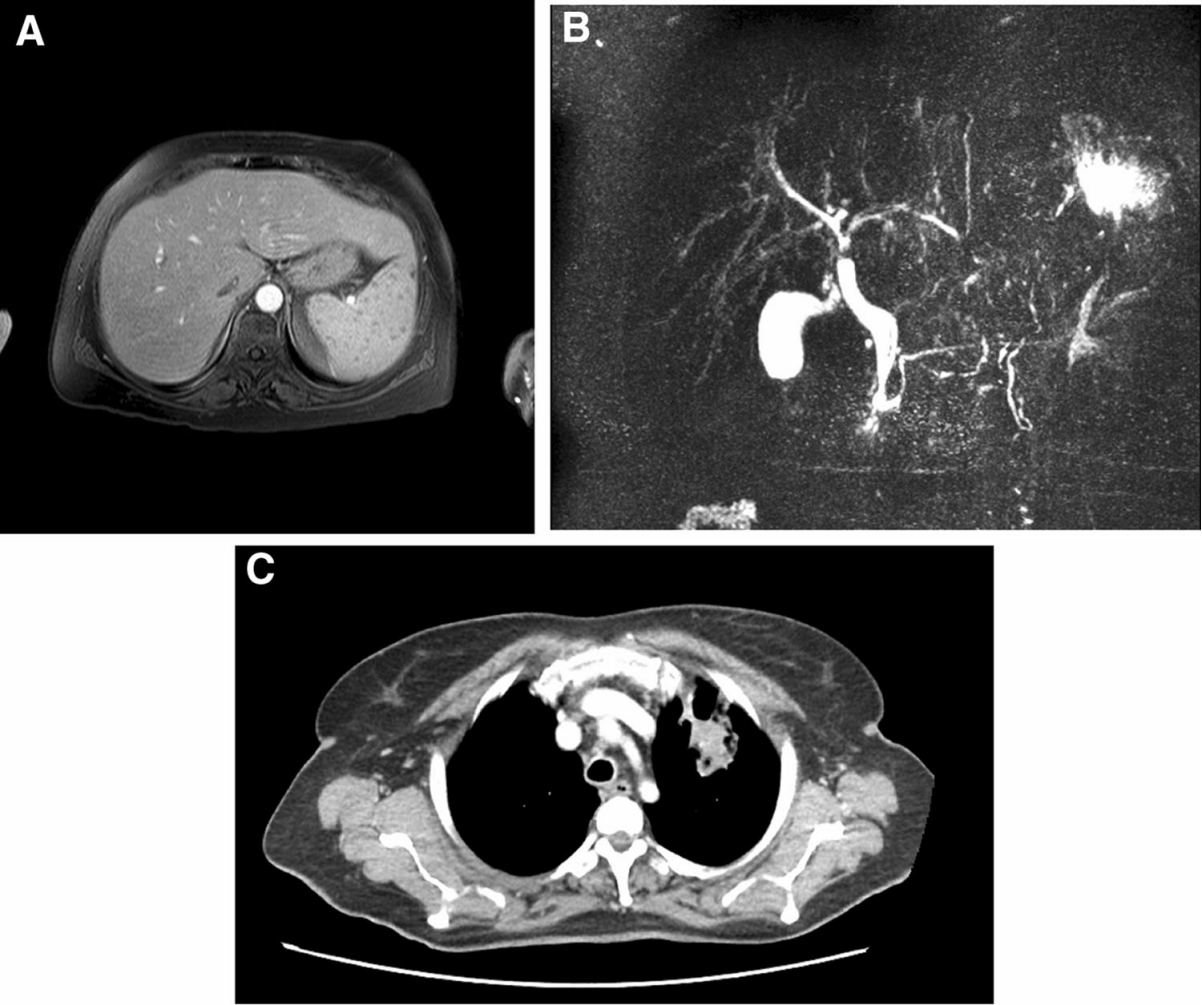
The magnetic resonance imaging shows multiple splenic lesions (**a**) and reduction of caliber of the proximal extrahepatic bile duct with ectasia of bile duct downstream (**b**) at disease onset. One month later, a new total body computerized tomography describes a dense tissue (diameter of 3 cm) with irregular margins infiltrating the upper lobe of the left lung, associated with ipsilateral hilar-mediastinal lymphadenopathy (**c**)

The patient underwent a magnetic resonance imaging (MRI), which revealed spleen lesions with hemangioma aspects, and highlighted a localized reduction of caliber of the proximal extrahepatic bile duct with ectasia of bile duct downstream (Fig. 1a, b). The endoscopic retrograde cholangio-pancreatography (ERCP) confirmed a compression ab estrinseco in the proximal tract of the bile duct, therefore, a prophylactic sphincterotomy was performed.

Positron emission tomography (PET) described some areas with a mild rise of glucose metabolism in the spleen and an important hyperaccumulation of radiopharmaceutical throughout hepatic parenchyma. One month later, clinical and laboratory findings did not change. The patient underwent a colonoscopy that revealed a normal mucosa, while a new CT described a dense tissue with a diameter of about 3 cm and irregular margins infiltrating the upper lobe of the left lung, associated with ipsilateral hilar-mediastinal lymphadenopathy (Fig. 1c). Cytology, on brushing and bronchoalveolar lavage by bronchoscopy, revealed bronchial epithelia, neutrophils and macrophages, while the presence of fungi and germs, including mycobacteria, was excluded. Peripheral blood lymphocytes immunophenotyping highlighted a severe reduction in CD8 T-cells. Spirometry pointed up a mild obstructive deficit with prevalent impairment of distal airways.

**Fig. 2:**
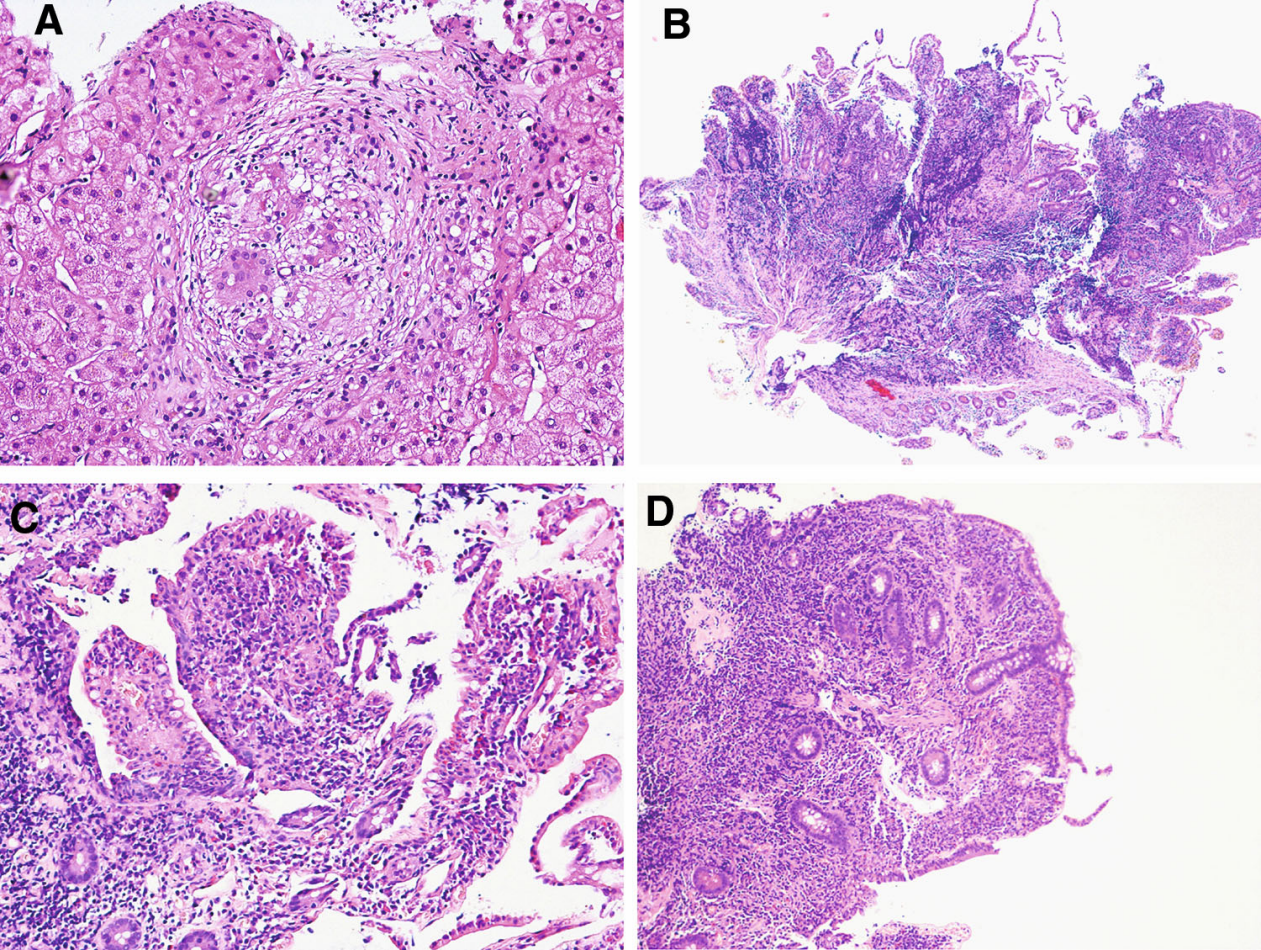
**a** Hematoxylin and eosin staining of liver tissue, showing a sarcoid, non-caseating granuloma, with the presence of typical epithelioid and giant cells (magnification ×20). **b**–**d** Hematoxylin and eosin staining of ileum mucosa, showing a lymphoplasmacytic and granulocyte infiltrate, with erosions, emperipolesis, glandular atrophy and distortion, and nodular lymphoid aggregates in the ileum mucosa (magnification ×2, ×10 and ×10, respectively)

Since the persistence of cholestasis (high serum levels of AP and γ-GT), expression of hepatic involvement in the course of a likely systemic disease, a liver biopsy was performed, revealing a sarcoid, non-caseating granulomatous process, with the presence of typical epithelioid and giant cells (Fig. 2a).

Methylprednisolone was administered (1.0 mg/kg/day) for 4 weeks and subsequently the dosage was reduced to 0.5 mg/kg/day.

**Fig. 3:**
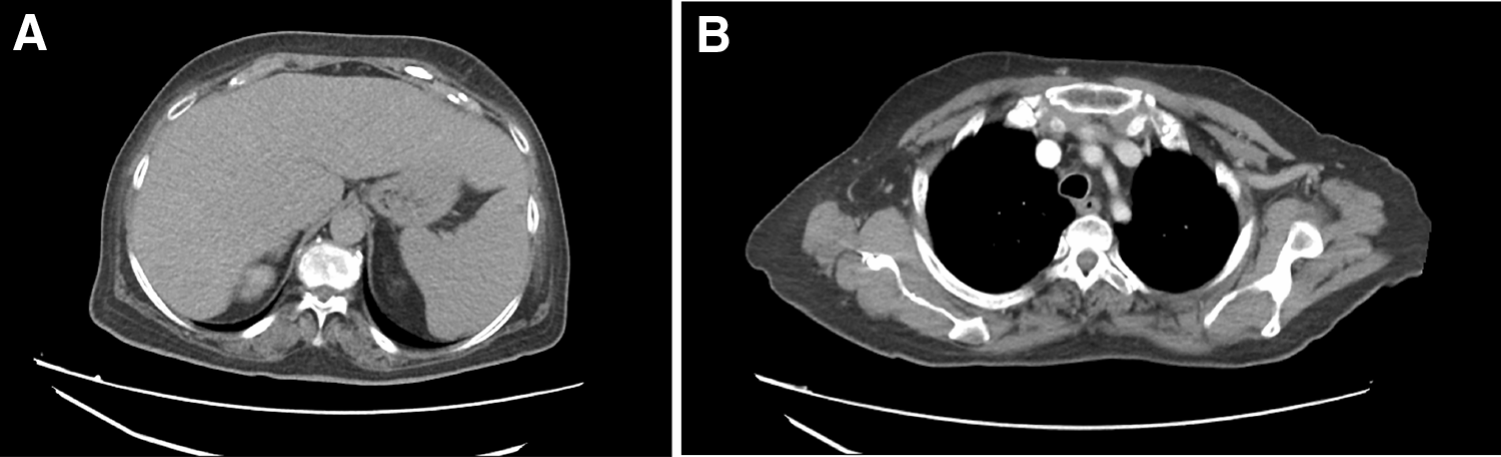
The computerized tomography shows a complete resolution of the splenic lesions (**a**) and of the lung infiltration (**b**)

Two months later, the patient showed a marked clinical improvement with the normalization of inflammatory and cholestasis indicators. Moreover, CT revealed a complete resolution of lung and splenic lesions (Fig. 3a, b). Therefore, methylprednisolone was further reduced to 0.25 mg/kg/day.

Six weeks later, the patient was again hospitalized for chronic diarrhea, with more than 10 watery stools daily. On physical examination she presented spontaneous diffuse abdominal pain. Widal–Wright serodiagnosis, parasitological and microbiological examination of stool, antibodies for celiac disease and test for lactose intolerance resulted negative; total IgE and IgA, thyroxine, gastrin and vasoactive intestinal peptide were in the normal range. Esophagogastroduodenoscopy and a new colonoscopy were performed with random biopsies. Macroscopically, gastroduodenal and colonic mucosa was normal, while the histological examination revealed a nonspecific very mild lymphoplasmacytic inflammatory infiltrate of gastroduodenal mucosa, but most notably the ileum mucosa was affected by an important lymphoplasmacytic and granulocyte inflammatory infiltrate, with erosions, emperipolesis, glandular atrophy and distortion, and nodular lymphoid aggregates in (Fig. 3b–d).

Methylprednisolone therapy was restored to 1.0 mg/kg/day and a marked improvement of diarrhea was achieved in a few days.

## Discussion

Sarcoidosis is a systemic disease which can affect different organs and tissues, but more than 90% of patients have pulmonary and mediastinal lymph nodes involvement at onset, while an extrapulmonary isolated localization is rare (less than 10%) [4]. Our patient’s first manifestation was a hepatosplenic involvement, since she initially showed only altered liver function tests and infiltrative lesions of the spleen at imaging investigations, with subsequent demonstration of typical non-caseating granulomas at liver biopsy.

Hepatosplenic localization is frequent in the systemic sarcoidosis, indeed more than 35% of cases shows altered liver tests [5, 6]. The spleen results infiltrated in 40% of autopsies and in 10% of imaging investigations. However, most of these patients have no symptoms, except for 5–7% of them [7, 8]. Furthermore, only rare cases of isolated hepatosplenic or splenic sarcoidosis have been reported [4, 7, 9, 10]. In line with these observations, our patient presented pulmonary involvement, in the form of infiltrative lesion of the left upper lobe with ipsilateral hilar-mediastinal lymphadenopathy, one month after disease onset. The question remains whether the hepatosplenic involvement would have been the lonely clinical manifestation if corticosteroid treatment had been administered earlier.

A peculiarity of our case report was the appearance of chronic watery diarrhea associated with diffuse abdominal pain a few weeks after methylprednisolone dosage reduction (0.25 mg/kg/day), despite the complete resolution of lung and splenic lesions and the normalization of inflammatory and cholestasis indicators.

Gastrointestinal (GI) involvement has been reported in less than 1% of patients with sarcoidosis, but the real incidence of this manifestation may be underestimated because it is rarely symptomatic [1]. Indeed, subclinical GI tract sarcoidosis has been evidenced in 5–10% of patients with systemic disease [11–13]. The stomach (above all gastric antrum) is the most frequently involved in GI sarcoidosis [14], presenting as a subclinical, ulcerative, or infiltrative gastric sarcoidosis [15–21]. Whilst, small and/or large bowel involvement represents the least common form [22–25]. In contrast with these observations, our patient showed a very mild nonspecific lymphoplasmacytic inflammatory infiltrate of gastroduodenal mucosa and an important inflammatory infiltrate of ileum mucosa with typical aspects of Crohn’s disease. In fact, the histological analysis of terminal ileum biopsies showed lymphoplasmacytic and granulocyte infiltrate, with erosions, emperipolesis, glandular atrophy and distortion, and nodular lymphoid aggregates. It is known that GI sarcoidosis can appear clinically and pathologically like a Crohn’s disease, a Whipple’s disease, or an infection by tuberculosis, syphilis or fungi. Especially colon and terminal ileum sarcoidosis could easily mime a Crohn’s disease, also in the histopathological findings [26–28]. Therefore, it is necessary to find the presence of non-caseating granulomatous inflammation to differentiate GI sarcoidosis from Crohn’s disease. However, it has been very rarely described as an association between sarcoidosis and Chron’s disease [29, 30], as well as three cases of sarcoidosis developed after Crohn’s disease treatment with natalizumab or infliximab [31, 32]. In our patient no granuloma was found, therefore, we should talk about a case of Crohn’s disease overlap, although we believe that the presence of sarcoid granulomas cannot be excluded 100%, because probably the GI sarcoidosis begins with lymphoplasmatycic proliferation, first organizing in nodular aggregates, and subsequently in granulomas. Moreover, the fast clinical improvement after high dosage steroid therapy is more typical of sarcoidosis. In conclusion, this case confirms that sarcoidosis is a pathology characterized by a wide variability of clinical presentation, tissues involvement and evolution, since our patient presented an isolated hepatosplenic localization at onset with a consequent atypical lung involvement, and finally, during an apparent recovery stage, she presented a plausible inflammatory bowel disease. Therefore, an intestinal inflammatory involvement should be seriously considered when the patient experiences symptoms.
